# PyGNA: a unified framework for geneset network analysis

**DOI:** 10.1186/s12859-020-03801-1

**Published:** 2020-10-22

**Authors:** Viola Fanfani, Fabio Cassano, Giovanni Stracquadanio

**Affiliations:** School of Biological Science, The University of Edinburgh, Edinburgh, EH9 3BF UK

**Keywords:** Geneset Network Analysis, Biological Networks, Network analysis workflow

## Abstract

**Background:**

Gene and protein interaction experiments provide unique opportunities to study the molecular wiring of a cell. Integrating high-throughput functional genomics data with this information can help identifying networks associated with complex diseases and phenotypes.

**Results:**

Here we introduce an integrated statistical framework to test network properties of single and multiple genesets under different interaction models. We implemented this framework as an open-source software, called Python Geneset Network Analysis (PyGNA). Our software is designed for easy integration into existing analysis pipelines and to generate high quality figures and reports. We also developed PyGNA to take advantage of multi-core systems to generate calibrated null distributions on large datasets. We then present the results of extensive benchmarking of the tests implemented in PyGNA and a use case inspired by RNA sequencing data analysis, showing how PyGNA can be easily integrated to study biological networks. PyGNA is available at http://github.com/stracquadaniolab/pygna and can be easily installed using the PyPi or Anaconda package managers, and Docker.

**Conclusions:**

We present a tool for network-aware geneset analysis. PyGNA can either be readily used and easily integrated into existing high-performance data analysis pipelines or as a Python package to implement new tests and analyses. With the increasing availability of population-scale omic data, PyGNA provides a viable approach for large scale geneset network analysis.

## Background

The availability of high-throughput technologies enables the characterization of cells with unprecedented resolution, ranging from the identification of single nucleotide mutations to the quantification of protein abundance [1]. However, these experiments provide information about genes and proteins in isolation, whereas most biological functions and phenotypes are the result of interactions between them. Protein and gene interaction information are becoming rapidly available thanks to high-throughput screens [2], such as the yeast two hybrid system, and downstream annotation and sharing in public databases [3, 4]. Thus, it is becoming obvious to use interaction data to map single gene information to biological pathways.

Integrating interaction information with high throughput experiments has proven challenging. The vast majority of existing analytical methods are based on the concept of over-representation of a candidate set of genes in expert curated pathways or networks [5, 6]; however, this approach is strongly biased by the richer-get-richer effect, where intensively studied genes are more likely to be associated with a pathway [7], ultimately limiting the power of new discoveries. Many methods have now been proposed to directly integrate network information for function prediction [8–10], module detection [11], gene prioritization [12] and structure recognition [13]. However, results are usually sensitive to the underlying network interaction model used and test statistics [14], and performing analyses across different tools is not feasible, as the vast majority of this software comes either as a web application or visualization plugins. While web applications are simple to use for targeted analyses, they are also difficult to integrate in high-throughput data analyses pipelines.

With the increasing availability of biological interaction resources and the development of standardized high-throughput analysis pipelines, a unified and easy to use framework for network characterization of genes and proteins could generate useful information for downstream experimental validation.

**Fig. 1:**
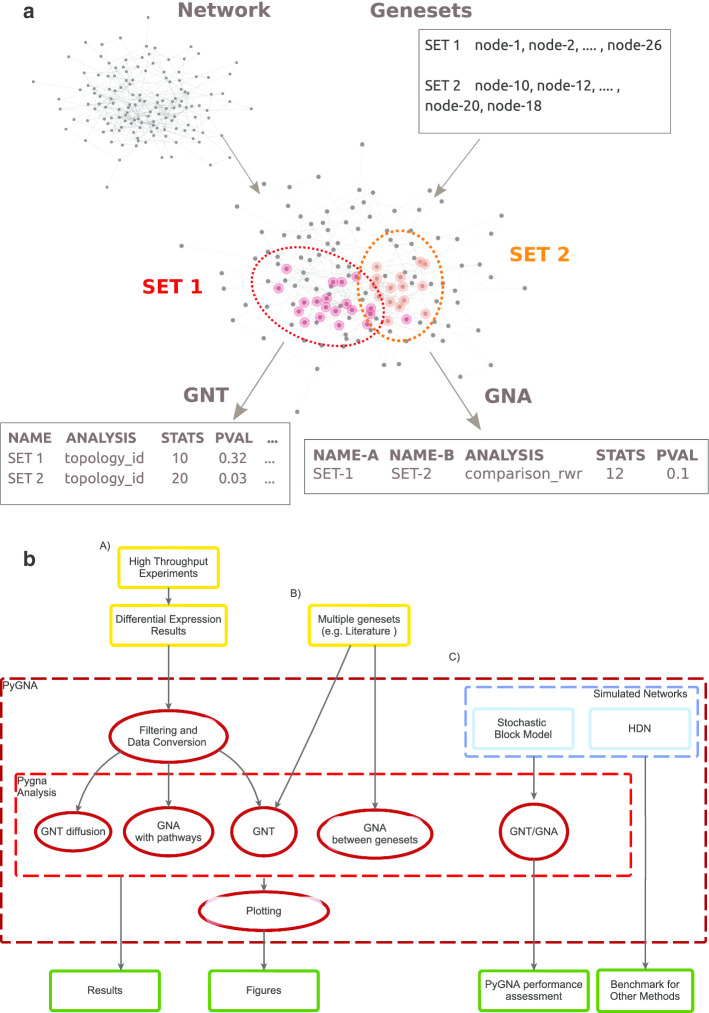
The PyGNA analysis workflow. **a** Outline of the GNT and GNA tests. Given an input network, PyGNA maps genes to network nodes, performs GNA and GNT tests, and then outputs the results in CSV format. **b** Complete workflow. We recognize three main use-cases where PyGNA can be used, including (i) network analysis of high-throughput experiments, (ii) network analysis of curated genesets and iii) simulations of networks and genesets for algorithms benchmarking. PyGNA can perform GNT analysis on single or multiple genesets, along with GNA analysis to identify network associated with other genesets or pathways. Results are provided as CSV files and as high quality PDF figures

Here we build on recent advances in network theory to provide an integrated statistical framework to assess whether a set of candidate genes (or geneset) form a pathway, that is genes strongly interacting with each other. We then extended this framework to perform comparisons between two genesets to find similarities with other annotated networks, as a way to infer function and comorbidities. We called our statistical tests geneset network topology (GNT) and geneset network association (GNA) tests, respectively (Fig. 1a). We implemented our tests into a Python package, called Python Gene Network Analysis (PyGNA). It is important to note that the tests implemented in our software are not an exhaustive list of all the approaches presented in literature; here we favoured well established models with test statistics easy to interpret [14]. Nonetheless, PyGNA provides a flexible API to implement and benchmark new network-based statistical tests, while taking advantage of our data processing and statistical testing framework.

We tested the GNT and GNA tests implemented in PyGNA on synthetic datasets to assess the performance (true positive rate and false positive rate). We then present how to use PyGNA to analyse high-throughput RNA sequencing data generated by The Cancer Genome Atlas (TCGA, [15]) and how to interpret network analysis results.

PyGNA is released as an open-source software under the MIT license; source code is available on GitHub (http://github.com/stracquadaniolab/pygna) and can be installed either through the PiP or Anaconda package managers, and Docker. Our software is designed with modularity in mind and to take advantage of multi-core processing available in most high-performance computing facilities. PyGNA facilitates the integration with workflow systems, such as Snakemake [16], thus lowering the barrier to introduce network analysis in existing pipelines.

The manuscript is organized as follows; “Methods” section describes the statistical network framework implemented in PyGNA, whereas  “Implementation” section describes PyGNA APIs and command line interface (CLI) options. In “Results” section, we present benchmarking results on simulated data and how to apply PyGNA to analyse RNAseq experiments. We conclude by discussing how PyGNA compares to other existing tools and why it represents an advancement for geneset network analysis.

## Methods

We hereby introduce basic notation and properties for network analysis, describing interaction models, test statistics and hypothesis testing methods implemented in PyGNA.

Let $G=(V,E)$ be a network, or graph, with |*V*| nodes and |*E*| edges. Let *A* be a matrix $|V| \times |V|$, with $A_{ij} = 1$ if there is an edge between node *i* and *j* and 0 otherwise; we denote *A* as the adjacency matrix of the network *G*. We hereby consider only undirected graphs, thus the adjacency matrix is symmetrical $A_{ij} = A_{ji}$; however, all the tests we present can be applied to directed networks and weighted networks. Moreover, unless otherwise stated, we consider only the largest connected component (LCC) of the network; while this is not strictly necessary, distance measures are often not informative when computed over disconnected graphs. We denote as degree of a node *i*, deg(*i*), the number of edges associated with it. In this context, nodes represent genes or proteins, whereas edges the intervening interactions, e.g. physical, genetic interactions.

Let $S=s_1, \ldots , s_n$ be a geneset consisting of *n* genes, we want to quantify the strength of interaction between genes in the geneset (geneset network topology, GNT) and with genes in another geneset (geneset network association, GNA).

### Interaction models

We denote as interaction model, a function that quantifies the strength of interaction between any two nodes in a network. Here we introduce three interaction models with different properties and complexity.

A direct interaction model assumes that two nodes interact only if there is an edge between them; this is the most efficient model to evaluate as it requires only the inspection of the adjacency matrix.

Under a shortest path interaction model, instead, we assume that the strength of interaction between two genes is a function of their distance on a network *G*, that is closer genes are more likely to interact. Thus, we denote with $i \rightarrow j$ a path in *G* from node *i* to node *j*, whose length, $l_{ij}$, is the number of edges from *i* to *j*. We then quantify the strength of interaction between two genes, *i* and *j*, as the length of the shortest path from *i* to *j*, denoted as $s_{ij}$; w.l.o.g, shortest paths can be also computed over directed and weighted networks.

Finally, we introduce a probabilistic model of gene interactions, namely the Random Walk with Restart (RWR) model. Let *W* be a stochastic matrix inferred from the adjacency matrix *A*, the probability of reaching node *i* from node *j* after *k* steps is $(W^k)_{ij}$ [17]. However, for *k* big enough, the probability of interaction between nodes converges to a quantity proportional to the degree of the nodes, thus neglecting local structure information. We here instead consider a random walk with restart model (RWR), where it is possible to return to the starting node with fixed probability $\beta$ (set to 0.85 unless otherwise stated [18]). We can then estimate analytically the probability of interaction at steady state as follows:

1 $$\begin{aligned} H = \beta (I - (1 - \beta ) {\bar{A}} )^{-1} \end{aligned}$$

where ${\bar{A}}$ is the normalized adjacency matrix obtained as ${\bar{A}} = A D^{-1}$, with *D* being the diagonal matrix of node degrees. In this case, the matrix *H* can be interpreted as the heat exchanged between each node of the network [11]. It is also worth noting that the above formulation is agnostic to direction and weights of the edges.

These three interaction models capture different topological properties. Direct models provide information about the neighborhood of a gene and its observed links. However, they might not be sufficiently powered to detect mid- and long-range interactions, thus statistics defined under these models are usually sensitive to missing links. Conversely, modelling gene interactions using shortest path provides a simple analytical framework to include local and global awareness of the connectivity. However, this approach is also sensitive to missing links and small-world effects, which is common in biological networks and could lead to false positives [19]. Propagation models provide an analytical model to overcome these limitations, and have been shown to be robust for biological network analysis [20]. While its interpretation is not necessarily straightforward, the RWR model is more robust than the shortest path model, because it effectively adjusts interaction effects for network structure; it rewards nodes connected with many shortest paths, and penalizes those that are connected only by path going through high degree nodes.

Based on the above interaction models, we have implemented and tested different statistics, which are described in detail below.

### Geneset network topology statistics

Let $S = {s_1, \ldots , s_n}$ be a geneset of *n* genes, each mapped to a node in $G=(V,E)$. We are interested in testing whether the strength of interaction between nodes of the geneset is higher than expected by chance for a geneset of the same size.

Under a direct interaction model, the importance of a geneset *S* can be quantified as the number of edges connecting each node in *S* to any other node in the network; we refer to this quantity as the total degree of the node. Thus, we define the total degree statistic for a geneset *S* as:

2 $$\begin{aligned} T_{TD} = \frac{1}{n} \sum _{i \in S} deg(i) \end{aligned}$$

While $T_{TD}$ could be helpful to have an idea of how relevant and well characterized the nodes in the geneset are, we do not expect this statistic to be informative on the strength of interaction withing a geneset.

Conversely, with the direct interaction model, the strength of interaction for a geneset *S* can be quantified as the number of edges connecting each node in *S* to any other node in the geneset; we refer to this quantity as the internal degree of the node. Thus, we define the internal degree statistic for a geneset *S* as:

3 $$\begin{aligned} T_{ID} = \frac{1}{n} \sum _{i \in S} \dfrac{deg(i,S)}{deg(i)} \end{aligned}$$

where *deg*(*i*, *S*) is the internal degree of gene *i* in geneset *S*. In practice, the internal degree statistic captures the amount of direct interactions between genes in a geneset, and thus a geneset showing a network effect should have $T_{ID}$ values close to 1. However, the main limitation of this model lies in the fact that it only captures direct interactions, whereas biological networks are usually characterized by medium and long range interactions.

Another way to assess the strength of a network effect is the size of the largest connected components of the graph induced by the geneset *S*, hereby denoted as $T_M$. A main concern regarding direct interaction methods is that they could fail in presence of missing links, which is a well-known problem in biological networks analysis, where experimental screens are often not sensitive enough to detect all existing gene/protein interactions.

A shortest path interaction model allows to overcome this limitation by explicitly taking into account the distance between nodes. Here we define the test statistic $T_{SP}$ for the geneset *S* as follows:

4 $$\begin{aligned} T_{SP}(S) = \dfrac{1}{n} \sum _{i=1}^n \min _{j\in S} s_{ij} \end{aligned}$$

which is the average of the minimum distance between each gene and the rest of those in *S* [21].

Conversely, under a RWR model, we can consider $h_{ij}\in H$ as the heat transferred from node *i* to node *j*, which can be used as a measure of interaction strength between the nodes in the geneset *S*, as follows:

5 $$\begin{aligned} T_{H}(S) = \sum _{i,j \in S, i\ne j} h_{ij} \end{aligned}$$

### Geneset network association statistics

Let $S_1$ and $S_2$ be two geneset with *n* and *m* genes respectively, we want to estimate the association between $S_1$ and $S_2$ as a function of the strength of interaction between their nodes.

Under a shortest path model, the association statistics $U_{SP}$ is defined as follows:

6 $$\begin{aligned} U_{{SP}} (S_{1} ,S_{2} ) & = \frac{1}{{n + m}}\sum\limits_{{i \in S_{1} }} {\min _{{j \in S_{2} }} } s_{{ij}} + \sum\limits_{{j \in S_{2} }} {\min _{{i \in S_{1} }} } s_{{ij}} {\text{ }} \\ &\quad - \frac{1}{2}(T_{{SP}} (S_{1} ) + T_{{SP}} (S_{2} )) \\ \end{aligned}$$

whereas, under a RWR model, we measure association as a function of the heat, $U_{H}$, transferred between the two genesets as follows:

7 $$\begin{aligned} U_{H}(S_1, S_2)= \sum _{i \in S_1, j \in S_2} h_{ij} + h_{ji} \end{aligned}$$

where we consider also the heat withhold by a gene, when there are overlapping genes between $S_1$ and $S_2$.

### Hypothesis testing

The topological and association statistics are ultimately used for hypothesis testing. To do that, we need a calibrated null distribution to estimate whether the observed statistics are more extreme than what expected by chance. Closed form definition of null distributions is possible only for very simple network models, which are often unrealistic. Therefore, we reverted to a bootstrap procedure to estimate null distributions of the test statistics, conditioned on the geneset size; while this approach can be computationally taxing, in practice, we observed that $\approx 500$ bootstrap samples are sufficient to obtain a stable distribution (see Additional file 1).

Thus, w.l.o.g, let *Q* be the null distribution of the test statistic *q* estimated for a geneset of size *n*, and ${\bar{q}}$ the observed value. It is possible to derive an empirical p-value as follows:

8 $$\begin{aligned} P({\bar{q}} \ge Q ) = \dfrac{(\sum _{i=1}^{|Q|}I(Q_i \ge {\bar{q}}))+1}{|Q|+1} \end{aligned}$$

where *I* is the indicator function returning 1 if and only if the evaluated condition is true, and unit pseudo-count is added for continuity correction. It is straightforward to adapt this formula to the case of testing whether a test statistic is smaller than expected by chance.

The default sampler generates null distributions by sampling nodes uniformly at random. However, certain metrics might be particularly sensitive to local network structure, especially when they solely rely on degree-related statistics to characterize a geneset. To overcome this problem, we also implemented an additional sampler that generates null distributions matching the degree distribution of the tested dataset.

For the GNA tests, it is important to note that we are now dealing with two genesets. Hence, a null distribution can be computed either by sampling two random genesets or by sampling only one of the two; we recognize that the latter is more conservative, and is recommended when checking for association with known pathways (see Additional file 1).

### Benchmarking geneset network tests

Rigorous benchmarking of network analyses tools is challenging, because there is no ground truth for geneset network analysis [14].

Stochastic block models (SBM) have been shown to be a reasonable model for analyzing biological networks [22]; importantly, since SBM define a generative process over networks, they can be used to create networks with controllable features, including modules (also often referred as clusters). Let $M: k\times k$ be a stochastic block model with *k* blocks, where $M_{ij}$ represent the probability of a node in block *i* to be connected to (or interact with) a node in block *j*. A new network with *n* nodes can be generated by assigning each node to a block and adding edges probabilistically using the block model matrix. It is straightforward to note that if $M_{ii}>> M_{ij}$ for any *j*, the genes in block *i* are likely to show a network effect. Hence, by modulating the values on the diagonal of the block model matrix, we can assess the performance of GNT tests by analyzing the genesets made of the genes in a block. Conversely, we expect to find a significant association between two blocks *i* and *j* if $M_{ij}>> M_{kl}$, with ${i,j} \ne {k,l}$. By parametrizing the off-diagonal terms of the block model matrix, it is possible to assess the performance of GNA tests (see Additional file 1 for a graphical representation of the SBMs).

While the SBM are useful to simulate networks with controllable structures, they are difficult to adapt to modelling networks with highly connected nodes (hubs), which are common in biological networks. Thus, here we introduce a stochastic generation procedure to build networks with hubs, which can then be used for assessing the performances of GNT tests. We hereby describe each model in detail.

#### SBM for GNT benchmarking

We use the SBM framework to simulate a network with *k* blocks, with a baseline probability of interaction within and between blocks, $p_0$. We then select $k^+ < k$ blocks from the SBM matrix and set their within probability of connection $M^+_{ii} = \alpha p_0$, where $\alpha > 1$ is a scaling factor controlling the strength of interaction of the genes within block *i* compared to the rest of the genes in any other block. Intuitively, each of the $k^+$ blocks represents a geneset with a significant network effect, thus a robust GNT test should be able to detect them.

Ultimately, by varying the size of highly connected blocks, the baseline probability of interaction $p_0$ and the strength of interaction $\alpha$, it is possible to assess the power, true positive rate (TPR) and false positive rate (FPR) of GNT tests under different conditions.

#### SBM for GNA benchmarking

Similar to the approach outlined for GNT benchmarking, we used the SBM framework to generate network with multiple gene clusters to assess the performance of GNA tests.

We use the SBM framework to simulate a network with *k* blocks, with a baseline probability of interaction within and between blocks, $p_0$. We then selected $k^+$ blocks at random and set their within block connection probability to $M^+_{ii} = \alpha p_0$ and their between blocks connection to $M^+_{ij} = \gamma p_0$ for $i\ne j$ and $\alpha ,\gamma > 1$. We then reparametrize $\gamma$ as a function $\alpha$, in order to control the relationship between the within and between block connection probability. Let $\beta = \gamma / (\alpha - 1)$, we can set the between block connection probability as $M^+_{ij}= p_0 + \beta p_0 (\alpha -1)$. With this parametrization, we can directly simulate 3 different scenarios:

1. if $\beta = 0 \Rightarrow M^+_{ij} = p_0$, the connection probability between blocks is equal to the baseline, thus genes in a block are highly connected.
2. if $0< \beta< 1 \Rightarrow p_0< M^+_{ij} < M^+_{ii}$, then the connection probability between the blocks is higher than the baseline, and thus we obtain assortative genesets.
3. $\beta> 1 \Rightarrow M^+_{ij} > M^+_{ii}$, then we have non assortative genesets, thus we expect them to be detected by a GNA test.

After building a network, we then generate genesets by selecting two distinct blocks, *i*, *j*, with *m* nodes each, and add $\pi \times m$ nodes from block *i* and $(1-\pi ) \times m$ nodes from block *j*; for simplicity, we picked genes from blocks containing the same number of genes. The GNA testing is then performed between the SBM blocks and the novel mixture blocks. By varying the size of highly connected blocks and their interaction probability, along with the geneset composition, it is possible to assess the true positive rate (TPR) and false positive rate (FPR) of GNA tests.

#### High degree nodes model for GNT benchmarking

The high degree nodes (HDN) model generates networks with a controllable number of hubs, $n_{hd}$, whose probability of connection with another node, $p_{hd}$, is higher than the baseline probability $p_0$ assigned to any other node in the network. The model is fully specified by four parameters, namely the number of nodes in the network, *n*, the number of HDN nodes, $n_{hd}$, the baseline connection probability, $p_0$, and the HDN connection probability, $p_{hd}>p_0$.

In order to benchmark GNT tests in presence of HDN nodes, we created geneset as a mixture of HDNs and non HDN nodes; we denoted these genesets as extended genesets. Specifically, each geneset is made of $\pi _{hd} \times n_{hd}$ nodes, with $\pi _{hd}\in (0, 1]$, and $\rho \pi _{hd} n_{hd}$ random high degree nodes, where $\rho$ is the ratio between high degree nodes and other nodes in the network (see Additional file 1 for a graphical representation).

With the HDN model, we can replicate a common scenario where the tested geneset is made of a few master regulators and many, possibly, unrelated genes. Here, the idea is that a robust GNT test should have a low false positive rate, even when observed statistics might be skewed by few highly connected nodes.

## Implementation

PyGNA is implemented as a Python package and can be used as a standalone command-line application or as a library to develop custom analyses. In particular, our framework is implemented following the object oriented programming paradigm (OOP), and provides classes to perform data pre-processing, statistical testing, reporting and visualization. Here we provide an overview of the package structure and available interfaces, although the complete API documentation is available at: https://github.com/stracquadaniolab/pygna. Our basic workflows are summarized in Fig. 1b.

### Input/output functions

Our software can read genesets in Gene Matrix Transposed (GMT) and text (TXT) format, while networks can be imported using standard Tab Separated Values (TSV) files, with each row defining an interaction. For diffusion analysis, instead, we require a Comma Separated Value (CSV) file specifying weights for each gene. It is important to note that parsers for new data can be easily implemented by extending the ReadData abstract class.

To facilitate the integration in bioinformatics pipelines, e.g. downstream analysis of DESeq2 results [23], we implemented a Utility class to enable input filtering, gene name conversion and GMT file creation.

PyGNA stores results as CSV files, for downstream manipulation and sharing, although new formats can be supported by extending the Output class. It is important to note that performing tests on large networks using either shortest path or random walk models is computationally taxing. However, since the node pairwise metrics are dependent only on the network structure, they can be computed upfront as part of a pre-processing step. Here, we save matrices in Hierarchical Data Format (HDF5) format, using the pytables framework [24], for efficient matrix storage. On this point, we designed PyGNA to performs efficiently both on low-memory machines, using memory mapped input output, and high-performance computing environments, by loading matrices directly into memory.

### Analysis functions

The GNT and GNA analysis are implemented by the StatisticalTest, and the StatisticalComparison classes, respectively. It is important to note that PyGNA can be easily extended to use different test statistics by defining new Python functions; on this point, in our online documentation, we provide a complete example on how to build GNT tests based on closeness centrality of the nodes.

A bottleneck of our network analysis framework is the bootstrap procedure used to obtain a null distribution for hypothesis testing. However, the resampling procedure is a seamlessly parallelizable process, since each randomly sampled set of nodes is independent from the others; thus, we implemented a parallel sampler using the multiprocessing Python library, allowing the user to set the number of cores to use. If only one core is requested, the multiprocessing architecture is not set-up, sparing the overhead incurred by setting up a scheduler for running only one thread (see Additional file 1). It is important to note that, currently, Python 3.8 is required in order to process large matrices on multi-core CPUs.

### Visualization functions

PyGNA has been developed to generate high quality figures for each analysis and to export networks and genesets in standard formats compatible with graph visualization software, such as Cytoscape [25]. The visualization functions are implemented as part of the PygnaFigure class, which comes with sensible default parameters to maximize figures readability.

There are four main types of figures currently implemented in PyGNA, namely bar plots, point plots, heatmaps and volcano plots, to visualize to GNT and GNA results.

Barplots are used to plot the GNT results for a single statistic. For each geneset a red bar represents the observed statistic, whereas a blue one represents the average of the empirical null distribution. To denote significance of each test we annotate the plot with stars, according to the $-log_{10}(p{\text{-}}value)$. An example is presented in Fig. 4c, as part of our results.

Conversely, a dot plot can be used to summarize multiple tests for the same geneset. In order to show all the results in the same figure, the observed values are transformed in absolute normalized z-scores, such that all significant tests have z-score $> 0$ and are marked with a red dot. An example is discussed in Fig. 4a.

GNA results can instead be visualised on heatmaps, with the color gradients used to report the strength of association between two genesets. When an all-vs-all test is conducted, as in Fig. 5, a lower triangular matrix is shown, with stars denoting significance. If, instead, a M-vs-N test was conducted, a complete heatmap would be included in the plot.

Alternatively, volcano plots can be used to visualize one-vs-many GNA results, for testing a geneset against a large number of datasets (e.g. gene ontologies). The plot shows the normalized z-score on the x-axis and the $-log_{10}$ of the p-value adjusted to control the False Discovery Rate (FDR) on the y-axis. Significant results are shown with red crosses, whereas not significant associations are represented by blue dots. We also annotate the plot with the top 5 scoring terms. An example of this plot is presented in the Additional file 1.

We provide more detailed information and tutorials in our online documentation.

### Network properties

On top of the statistical testing framework, we provide functions for the basic characterization of the network and geneset. General information, such as number of nodes and edges, average degree, connected components of the graph can all be retrieved from command line and saved in textual formats or shown in a GraphML file.

### Network simulation functions

PyGNA provides a comprehensive simulation framework to generate networks with different structures and properties for benchmarking purposes, as described in “Benchmarking geneset network tests” section. Moreover, since we allow the user to implement further statistical tests, we provide a full pipeline to generate a benchmark dataset to compare the results with those available in this paper.

Model descriptions and implementation details are also available in our online documentation.

### Command line interface and workflow system integration

PyGNA implements a standard Unix-like command line interface with robust default options set for all functionalities. Using a CLI interface facilitates integration with workflow analysis systems, such as Snakemake [16]. We have developed Snakemake pipelines to perform network analysis, available at https://github.com/stracquadaniolab/workflow-pygna, which can be readily integrated into existing workflows.

## Results

We designed PyGNA as a tool to streamline network analysis of biological data. Here we perform an extensive analysis of the performances of the GNT and GNA tests implemented in PyGNA and then, we present a common use case regarding the analysis of cancer RNA sequencing (RNA-seq) experiments, providing basic guidelines to interpret PyGNA results.

### Network simulations and algorithm benchmarking

#### GNT benchmarking with SBM and HDN

We used the SBM and HDN network models to assess the performance of the GNT tests implemented in PyGNA.

**Table 1:** GNT and GNA benchmark parameters

Parameter	Description	GNT-SBM	GNT-HDN	GNA-SBM
*n*	Number of nodes	1000	1000	1000
*k*	Number of blocks	7	–	9
$p_0$	Baseline connection probability	0.01, 0.02, 0.05	0.006, 0, 02	0.01, 0.02
*m*	Size of the geneset	20, 50, 100	$9, \dots , 200$	50, 80
$\alpha$	Within block connection probability scaling	2, 3, 5, 10	–	2, 5
$\beta$	Between block connection probability scaling	0	–	0, 10
$p_{hd}$	HDN connection probability	–	0.5, 0.2, 0.1, 0.08, 0.05, 0.01	–

For each model, we report the name of the parameter, a short description and its setting. A dash is reported when the parameter is not used

To do that, we first generated networks using the SBM model using the parameters reported in Table 1 (GNT-SBM). Given the large number of parameters, we restricted our analyses to networks generated using $k=7$ blocks. For each network, we set $\lfloor k/2 \rfloor$ blocks with connection probability $\alpha p_0$ to simulate genesets with a network effect, which we denoted as positive genesets, whereas the remaining $k-\lfloor k/2 \rfloor$ were denoted as negative genesets. For each possible parameter setting, we generated 10 networks and corresponding genesets for a total of 5400 positive and 5400 negative genesets.

**Fig. 2:**
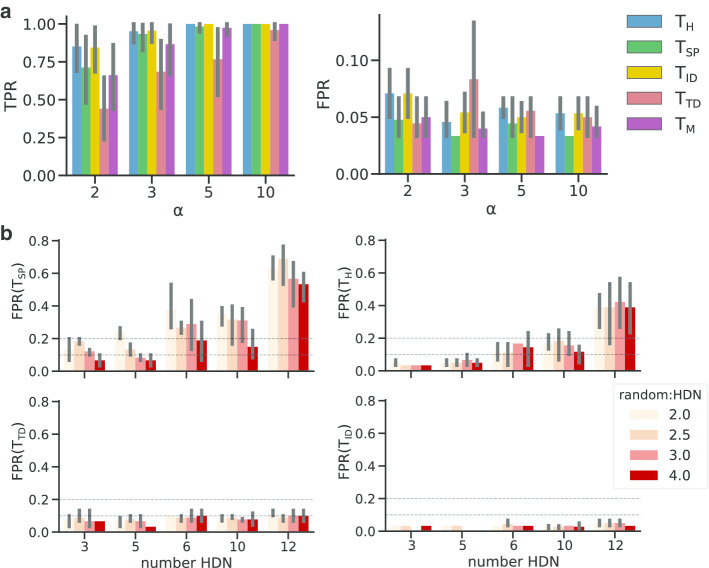
GNT benchmarking. **a** Performance of all GNT tests on the SBM networks. We show True Positive Rate (TPR) and False Positive Rate (FPR) (y-axis) of each GNT test (colors) for different values of $\alpha$ (x-axis). As expected, as the value of $\alpha$ increases, all tests improve their detection performance, with $T_H$ and $T_{ID}$ having consistently TPR $>0.75$. Conversely, for FPR we do not see a strong effect as $\alpha$ increases, with most tests having FPR $\sim 5\%$. **b** Extended geneset high degree nodes (HDNs) networks used to quantify FPR. Genesets have been selected with increasing number of HDNs (x-axis) and random nodes to HDNs ratios (colors); for each analysis, we report the False Positive Rate (FPR). As the ratio between random and HDNs increases ($\rho$), we notice that $T_{SP}$ has better performances. Interestingly, $T_{ID}$ is the only one with FPR $<5\%$ in all conditions

We found that $T_{ID}$, $T_{H}$ and $T_{SP}$ are the statistics with the best overall performances (Fig. 2a), with TPR $>70\%$ for all instances, whereas $T_{M}$ and $T_{TD}$ were able to detect a network effects only for highly connected genesets. In general, we found that all tests are robust to false positives ($FPR<10\%$ for all tests), with $T_{SP}$ being the most conservative.

We then used the HDN model to estimate the FPR of the GNT tests with respect to networks with hubs. Here we generated networks using the parameters reported in Table 1 (GNT-HDN) and generated 10 networks for each parameter setting. For each network, we then created extended genesets with by varying $\pi _{hd} = 0.1,0.2,0.5$ and $\rho =2,2.5,3,4$. For each combination of $\pi _{hd}$ and $\rho$ we generated 3 random genesets, for a total of 30 datasets for each combination of network and geneset parameters. It is important to note that for increasing $p_0$, $p_{hd}$ and $\pi$ values, the extended genesets begin to form connected clusters; these cannot be considered false positives, albeit being generated at random. Thus, for each geneset, we first computed the size of the largest connected component (LCC), and discarded those genesets with more than $75\%$ of the genes belonging to the LCC.

Here we found that our tests have a low FPR ($<10\%$) regardless of geneset composition and network structure. Interestingly, while $T_{SP}$ was the most robust on SBM networks, it is the most sensitive to HDN in the networks, with FPR as high as $20\%$ even for genesets with only 3 HDNs (Fig. 2b). In this case $T_{ID}$ is the most robust test (FPR$<10\%$), while $T_H$ has FPR$>0.2$ when the number of HDN increases.

Taken together, the $T_{ID}$ statistic is the one achieving the best performances and it is faster to compute respect to the other best performer, $T_{H}$, which requires the computation of a random walk matrix. Nonetheless, for exploratory analyses, we recommend using the $T_{H}$ test, which is confirmed to be well powered to detect network effects and has a low FPR, and might less sensitive to missing links. We would also point out that, since PyGNA provides implementations of the GNT analysis under different models of interaction, ensemble analyses could be useful in practice to increase the power of detecting network effects.

#### GNA benchmarking with SBM

We tested also the performance of GNA tests by generating networks and genesets as outlined in "SBM for GNA benchmarking" section and using the parameters reported in Table 1. For each network, we set two groups of blocks, $k^+ = 4$ and $k^-=4$, both of size *m*, along with another one including the remaining $N - k\times m$ nodes. We then set $M_{ij} = \gamma p_0$, for $i = 1,\dots , 7$ and $j=i+1$. For each pair of blocks, we generated genesets with a varying mixture of nodes $\pi = \{0.04,0.06,0.1,0.12\}$; with these genesets, we can test associations between highly connected and partially overlapping genesets. For each network and geneset parameter, we generated 10 runs, for a total of 2640 datasets. For both $U_{H}$ and $U_{SP}$, we then assessed the TPR, as the ratio of significant tests between genesets with $\beta = 10$, and the FPR, as the ratio of significant tests between genesets with $\beta =0$.

**Fig. 3:**
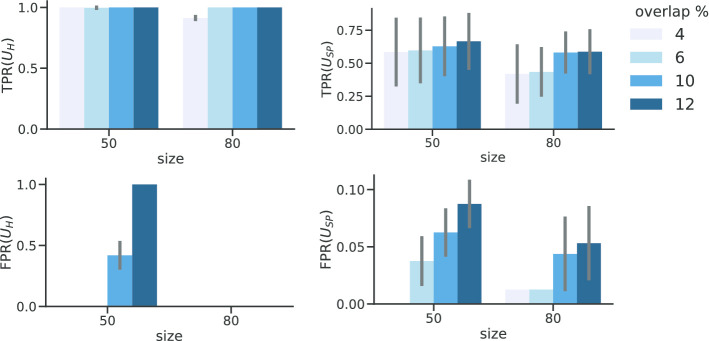
GNA benchmarking. **a** Performance of all GNA tests on SBM benchmark data. On the left column, we report the True Positive Rate (TPR) and False Positive Rate (FPR) for $U_H$, while on the right column we report the same metrics for $U_{SP}$. On the x-axis, we show different geneset sizes, while we denote the overlap between the tested genesets with colors. For example, for size 50 and $4\%$ of overlap the two geneset share 2 nodes. We notice that $U_{H}$ has TPR $>0.95$, while $U_{SP}$ is consistently below 0.75. Moreover, the FPR analysis confirms better performance for $U_H$, albeit it is skewed by many overlapping nodes. On this point, when two genesets share 6 or more nodes out of 50, $U_H$ always considers them as positives

We found that $U_H$ has higher TPR than $U_{SP}$, regardless of network structure and geneset composition (see Fig. 3). However, it is more prone to false discoveries when the number of overlapping nodes increases. In particular, when two genesets do not have high inter-connectivity, but share more than 5 out of 50 nodes the test is always significant. Importantly, all tests between non overlapping genesets are not significant.

Taken together, our results suggest that for $U_H$ is a well powered test for exploratory analyses, whereas $U_{SP}$ might be more appropriate for verifying known associations.

### Use case: network analysis of RNA sequencing experiments

RNA-seq experiments aim at finding genes that are up or down regulated between two or more conditions. As a use case, we analyzed RNA sequencing data generated by The Cancer Genome Atlas (TCGA) project [15] for 6 different types of cancer (see Additional file 1). Specifically, we selected 4 epithelial tumors, including 2 from urogenital tissues (BLCA and PRAD), 1 from breast (BRCA) and 1 from lung (LUSC), and 2 from liquid cancers (LAML and DLBC).

Here we are interested in finding whether differentially expressed genes in each cancer show a network effect, and whether they are similar to any other cancer analysed. It is possible to address these questions using the GNT and GNA tests implemented in PyGNA.

To do that, we retrieved TCGA data and performed differential expression analysis (DEA) using the TCGABiolinks package [26]. Here we found that there are no control samples in TCGA for LUSC, LAML, and DLBC; in this case, we instead used gene expression data from the Genotype-Tissue EXpression (GTEX) project [27], as control, and the TCGA tumor data processed by the Recount2 project [28], in order to avoid biases introduced by different RNA quantification pipelines (see Additional file 1). Taken together, we retrieved 6 datasets providing mRNA abundance for $\approx 15000$ genes for each tumor and performed differential expression analysis. For each dataset, we consider significant all genes with $FDR < 0.01$ and $|logFC|>3$ (see Additional file 1).

We then used PyGNA to perform GNT analysis and GNA analysis between all cancer datasets, using the BioGRID interaction network [29], a publicly available repository of protein interactions defining a human protein interaction network of 17331 nodes and 283991 edges. For each test, PyGNA returns the results as a CSV file, which includes descriptive statistics and the parameters of the null distribution used for hypothesis testing. Our workflows are summarized in Fig. 1 and Snakemake pipelines are available at: https://github.com/stracquadaniolab/workflow-pygna.

**Fig. 4:**
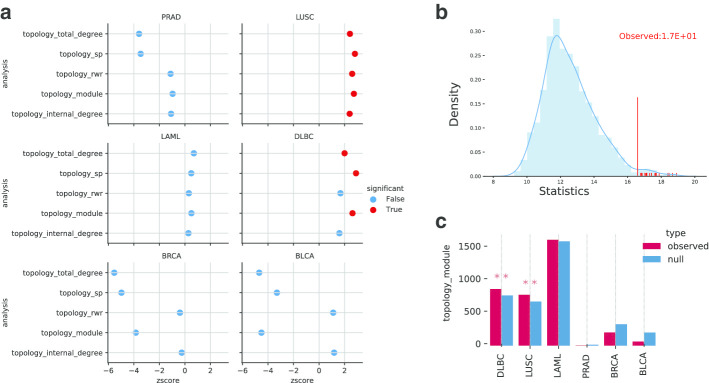
GNT analysis of TCGA RNA sequencing experiments. **a** Summary of the GNT results on the TCGA datasets. For each geneset analysed, a summary of all test results is reported. In order to make results comparable, observed test statistics are transformed in normalised z-scores. All results are in a scatter plot, where significant tests are marked with a red dot. We can notice that only the TCGA Lung Squamous Cell Carcinoma geneset is significant for all topology tests. **b** Null empirical distribution (blue) and observed value (red bar) for a significant rwr test on the TCGA Lung Squamous Cell Carcinoma geneset. **c** Barplot of the GNT module analysis on all TCGA datasets. For each geneset, we report both the observed statistic and the empirical null distribution average. Stars are used to identify significance of the test. Here, DLBC (p-value:$6.99 \times 10^{-3}$) and LUSC (p-value: $8.99 \times 10^{-3}$) are significant, while the other terms are not

We then used the PyGNA plotting tool (paint-summary-gnt) to visualize a summary of the GNT results for all datasets (Fig. 4a), where we report the test statistic as a z-score, to make them comparable across different tests. Interestingly, only differentially expressed genes in lung and lymphoid cancers show a significant network effect, albeit this is detected by all tests for lung cancer and only by $T_H$ and $T_{ID}$ lymphoid neoplasm. Interestingly, we did not observe any network effect for the other cancers; this could be explained by the fact these cancers might be controlled not by one highly connected network, but by multiple distinct ones.

We then used PyGNA diagnostic plot generated by the GNT analysis to visualize the effect size and the null distribution of the test statistic for one of our significant datasets; in Fig. 4b, the plot shows the observed value of the $T_{H}$ statistic for lung cancer (vertical red line) being located in the upper-tail of the null distribution (blue area), suggesting that a network effect has been detected.

We again used the PyGNA plotting tool (paint-datasets-stats) to present a summary of the GNT $T_M$ results for all datasets (Fig. 4c). For each geneset, we report both the observed statistic and the empirical null distribution average. Stars are used to identify significance of the test. Here, DLBC (p-value:0.00699) and LUSC (p-value: 0.00899) are the only cancers with a statistically size of the induced module.

**Fig. 5:**
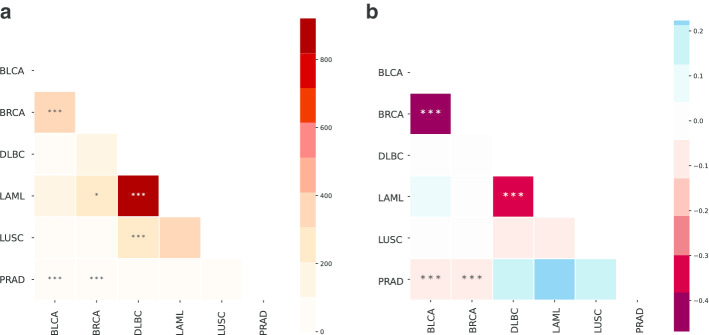
GNA analysis of TCGA RNA sequencing experiments. **a** Heatmap of the observed values of the GNA test under a RWR interaction model, $U_{H}$, where darker colors denotes larger observed $U_H$ values and stars denote statistical significance. **b** Heatmap of the observed values of the GNA test under a shortest path interaction model, $U_{SP}$. A divergent palette marks distant datasets with blue hues, and close ones with red hues, ($U_{SP} < 0$)

We then performed a GNA analysis between all differentially expressed genesets using the command (paint-comparison-matrix) in PyGNA. While most of them does not seem to show a consistent network effect, we can use the GNA to test whether each set of differentially expressed genes are more connected with each other than expected by chance. Using either $U_{H}$ and $U_{SP}$ tests, we found a significant association between breast, bladder, and prostate carcinomas, and between leukemia and lymphoid neoplasms (Fig. 5); this is clearly shown through darker gradients for strongly associated genesets, and by the star notation to report statistical significance. This result is consistent with other gene expression analyses, which have shown that anatomically related cancers or with similar histopathology share similar changes in gene expression [30]. Interestingly, we found a significant association between lung and lymphoid neoplasms; this might be explained by the fact that lungs contain a vast lymphatic network, which might also be dysregulated in lung tumors.

Taken together, we have shown how PyGNA enables network analysis of RNA sequencing datasets and provide useful biological insights. The availability of informative diagnostic and descriptive plots provides a simple entry point for downstream expert analyses.

## Discussion

The availability of biological interaction data has propelled the development of a plethora of network analysis methods, with the promise of linking single genes and protein information into networks to understand biological processes.

**Fig. 6:**
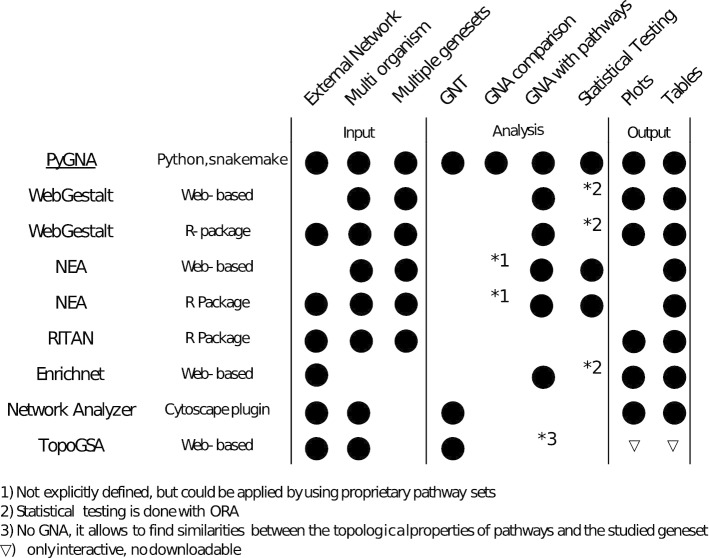
State-of-the-art tools for geneset network analysis. Comparison between publicly available, documented and actively maintained network analysis tools. For each tool, we reviewed the type of networks and genesets that can be given as input (e.g. multi-organism, external/custom defined), and whether a tool can generate tables and figures. The majority of tools provides only one type of network analysis, either GNT or GNA, with few of them providing association tests between multiple user defined genesets. We also noted that, for many tools, there are no statistical testing procedures. Conversely, PyGNA enables comprehensive statistical network analysis under different interaction models, testing both single geneset topology and multiple genesets association. Moreover, PyGNA takes input user-defined networks, regardless of their type and organism, and provides results in comma separated value (CSV) files and PDF figures

We surveyed publicly available, documented and actively maintained network analysis tools and found that, currently, PyGNA is the only available framework for comprehensive statistical network analysis under different interaction models (see Fig. 6). Currently, most software is available as web applications rather than stand-alone tools, usually performing only quantitative analyses with no statistical testing. This brings major limitations both for data interpretation and downstream integration into existing data analysis pipelines (e.g. RNA-seq and variant calling workflows); PyGNA directly addresses these problems, by implementing statistical analysis tools into a modular software package.

We further reviewed available tools by classifying their functionalities either as GNT or GNA, whether they perform statistical analysis and whether they provide a command line interface (CLI). We found two tools performing GNT on user defined genesets: TopoGSA [31] and NetworkAnalyzer [32]. TopoGSA is a web application implementing network topology geneset analysis. It evaluates topological properties of the subnetwork induced by an input geneset, such as average shortest path length, node degree and clustering coefficient. An empirical p-value is obtained through permutations, but the limited number of samples generated do not ensure a stable distribution for hypothesis testing. TopoGSA checks also for similarities with known pathways just by comparing network properties, but no statistical testing is performed, which ultimately limits its utility for interpreting the data. The application presents results in interactive tables and plots, and facilitate access to pre-computed networks of several organisms, along with the option to import user-defined networks. NetworkAnalyzer is a Cytoscape plugin, which estimates topology features of the subnetwork induced by a geneset, including centrality measures, average shortest path, node degree distribution. Differently from TopoGSA, it only provides descriptive statistics but no statistical analysis can be performed.

PyGNA instead provides robust topological statistical testing under different interaction models, which enables in depth analysis of the data, and represents a better solution for topology analysis.

Interestingly, we found GNA analysis to be a more popular application, in particular to study association with known pathways. The vast majority of tools perform association analysis using either over-representation analysis (ORA), which is usually a variant of Fisher’s exact test, or geneset enrichment analysis (GSEA, [33]). However, none of them explicitly allows association analysis between multiple user-defined genesets. There are three available tools commonly used for GNA analysis with known pathways: Webgestalt [34], network enrichment analysis (NEA, [35]), and Enrichnet [10].

Webgestalt is a comprehensive suite for geneset analysis, which implements conventional ORA and GSEA analysis, and performs association testing as network topology association (NTA) test using Gene2Net (http://www.gene2net.org/). First, a subnetwork is built from the input geneset by adding relevant neighbours using a random walk model, as implemented in NetWalker [36]. Then, the application performs ORA between the genes in the inferred subnetwork and a pathway databases. We found Webgestalt to be the most comprehensive tool for GNA, as it includes multiorganism and multiplatform support, interactive plots and downloadable results. NEA performs GNA by computing an enrichment score between an input geneset and a pathway, as a function of the number of edges shared between the two. The statistical significance of the score is assessed by randomly permuting the edges in the network; recently, a binomial test has been implemented to reduce the running time. NEA has been implemented both as an R package, NEArender [37], and as a web application, EviNet [38], which provides access to multiple network and pathway repositories (e.g. GO, KEGG, Biocarta). Enrichnet performs GNA analysis between a user-defined geneset and a predefined list of biological pathways. The application uses RWR to compute interaction probabilities between the input geneset and each pathway. The interaction probabilities are transformed into a score, $X_d$; intuitively, $X_d$ is a measure of how close the geneset is to the pathway compared to all the others. As the $X_d$ score does not allow a direct statistical testing, it is combined with Fisher-test FDR corrected p-values from an ORA, to find the threshold for significance. Enrichnet is a useful tool for direct comparison of ORA and a GNA, albeit it was last updated in 2012 and new pathways cannot be imported.

Since PyGNA provides also API for statistical network analysis, we also reviewed Ritan [39], an R package that provides functions for genesets and networks analysis in R. Ritan provides ORA testing between a geneset and pathways, provides functions to export networks for Cytoscape and iGraph, but it does not include any GNT or GNA off-the-shelf functionality. Finally, we would like to point out that the vast majority of GNA tests, are designed and optimized to perform association tests with specific datasets (e.g. KEGG pathways or Gene Ontology), rather than addressing the more general problem of network association; this poses substantial technical challenges for any rigorous benchmarking experiment based on synthetic networks.

Taken together, our software is the only available solution to easily investigate network properties under different interaction models and perform statistical testing. We recognize that web applications are easier to interact with, fast to use for small scale and targeted analyses, since they do not require any setup and integrate many network and pathway genesets. However, we have designed PyGNA with flexibility and scalability in mind; we provide both command line interface and open APIs to extend GNT and GNA analysis using different topology measures. Moreover, the support for multi-core processing and easy integration with Snakemake allows to run PyGNA on multiple datasets and experiments at a glance

## Conclusions

The availability of gene and protein interaction data provide unique opportunities to understand the cellular wiring underpinning most common complex phenotypes. However, integrating network and gene-level information has been challenging. Geneset network analysis provides a statistical framework to test the presence of interactions between genes associated with a phenotype, thus providing a useful tool for downstream analysis of high-throughput data. However, there are only few tools for statistical geneset network analysis, and usually are limited to specific interaction models, lack statistical testing methods or are only accessible through web applications.

Here we present a modular Python package, called Python Geneset Network Analysis (PyGNA), to perform statistical geneset network analysis under different interaction models. As networks analysis results are sensitive to the underlying gene and protein interaction model, it is important to perform these analyses using different models to gain confidence on the observed network effects. Different from existing applications, we designed PyGNA to be easily integrated into workflow systems and rapidly provide a comprehensive network characterization of input genesets. Our software takes advantage of multi-core architectures and can work both on desktop and high-performance computing environments, thus lowering the computational requirements to perform network analysis. Our software is available on GitHub (http://github.com/stracquadaniolab/pygna) and can be easily installed from PyPi, Anaconda and as a Docker container.

We have shown how PyGNA can be used as part of biological data analysis pipelines, in particular as downstream analysis tool for differential expression experiments, exploratory geneset analyses, and as a network simulation framework. It is also worth mentioning that, while the package development has been motivated by the need for an integrated tool for biological data analysis, ranging from RNAseq experiments to evolutionary genomics [40] PyGNA is agnostic to input data types and could easily be adopted to analyse non-biological networks, including social and communication networks, where the information can be summarized into sets of nodes (e.g. users of a Facebook group).

PyGNA is not only a stand-alone application, but also a Python library that can be easily integrated into other software; thus, we envision our framework as an open-source platform to develop network statistical tests.

## Availability and requirements

- Project name: PyGNA
- Project home page: https://github.com/stracquadaniolab/pygna
- Operating system(s): Platform independent
- Programming language: Python
- Other requirements: pandas, numpy, scipy, matplotlib, pyyaml, tables, seaborn, palettable, networkx, statsmodels, argh, mygene (Python 3.8 is required to use large matrix analysis on multiple processors)
- License: MIT license
- Any restrictions to use by non-academics: Not applicable

## Supplementary information

**Additional file 1**. contains all supplementary materials and figures referenced in the main manuscript. Section 1 1.1 describes more in depth th paralle sampling performance, Section 1 1.2 describes the stability of empirical null distributions, Section 1 1.3 describes the geneset network association bootstrapprocedures, Section 1 1.4 describes materials and preprocessing stepts for the TCGA data analysis. Section 2 is instead dedicated to the supplementary figures that are referenced in the main text.

## Data Availability

Data have been deposited on Zenodo and are freely accessible at: 10.5281/zenodo.3922015.

## Abbreviations

PyGNA: Python Geneset Network Analysis
GNT: Geneset network topology
GNA: Geneset network association
TCGA: The cancer genome atlas
CLI: Command line interface
LCC: Largest connected component
RWR: Random walk with restart
SBM: Stochastic block model
TPR: True positive rate
FPR: False positive rate
HDN: High degree nodes
OOP: Object oriented paradigm
BLCA: Bladder urothelial carcinoma
BRCA: Breast invasive carcinoma
PRAD: Prostate adenocarcinoma
LUSC: Lung squamous cell carcinoma
LAML: Acute myeloid leukemia
DLBC: Lymphoid neoplasm diffuse large B-cell lymphoma
DEG: Differentially expressed gene
GTEX: Genotype-tissue expression project
ORA: Over-representation analysis
