# Supplementary material for: PyGNA: a unified framework for geneset network analysis
Source: BMC Bioinformatics. 2020 Oct 22;21:476. doi: 10.1186/s12859-020-03801-1 (PMC7579948; doi:10.1186/s12859-020-03801-1)
Supplement: Supplementary file 1 — Additional file 1. contains all supplementary materials and figures referenced in the main manuscript. Section 1 1.1 describes more in depth th paralle sampling performance, Section 1 1.2 describes the stability of empirical null distributions, Section 1 1.3 describes the geneset network association bootstrapprocedures, Section 1 1.4 describes materials and preprocessing stepts for the TCGA data analysis. Section 2 is instead dedicated to the supplementary figures that are referenced in the main text. [file 12859_2020_3801_MOESM1_ESM.pdf]

SUPPLEMENTARY MATERIALS

PyGNA: a unified framework for geneset network analysis

Viola Fanfani, Fabio Cassano and Giovanni Stracquadanio*

Contents

1 Supplementary materials 1
1.1 Parallel sampling performance 1
1.2 Stability of empirical null distributions . 1
1.3 Geneset network association bootstrap procedures 1
1.4 TCGA data retrieval and preprocessing 2

2 Supplementary Figures 4

1 Supplementary materials

1.1 Parallel sampling performance

We tested how our sampler scales as a function of the number of cores allocated to PyGNA. We used the interaction network defined in [1] (13460 nodes, 138427 edges) and the generated genesets by taking random nodes from it; using a smaller network, allowed us to to minimize input/output overhead caused by reading HDF5 files. We then performed GNT analyses using both the module and random walk statistics, T_M and T_H, to test the performances when the statistic is estimated from a large matrix and when is evaluated only from the network structure. We performed our tests on genesets of size $[50, 100, 500]$ and by increasing number of cores $[1, 3, 6, 8]$ and permutations $[500, 1000, 10000]$ as shown in Fig. 1; experiments were performed on a Intel 3.2 GHz Intel Core i7 with 6 cores and 16Gb of RAM, running MacOS Mojave.

As expected, parallel sampling dramatically reduces the running time required to generate null distributions for the module test, although the maximum relative speedup was achieved when using 2 cores. For the T_H analysis, the most significant improvement was observed when running PyGNA on large genesets with more than thousands permutations. In general, for small genesets and a limited number of permutations, the cost of setting up the multiprocessing environment introduces a significant computational overhead; also, as expected, when allocating more than the number of available cores on the system, we observed no improvement or an increasing running time.

Taken together, we recommend using multiprocessing when large genesets are analysed or a large number of permutations are required to obtain a stable null distribution.

*Correspondence: giovanni.stracquadanio@ed.ac.uk
School of Biological Science, The University of Edinburgh, EH9 3BF Edinburgh, UK
Full list of author information is available at the end of the article

1.2 Stability of empirical null distributions

We determined experimentally the number of samples to be drawn to obtain a stable empirical null distribution for the GNT testing.

To do that, we used two real networks that we know have different densities and node degree distribution, namely the BioGRID network and smaller metabolic network reported by [1]. We then conducted our tests as follows: given a network G, we sample N_{gs} genes, which represent our tested geneset, and then apply GNT analysis with NoP number of permutations. For each scenario, we repeat the procedure R times, and record mean and standard deviation of the test statistic.

Here, we performed simulations for $N_{gs} = [50, 100, 200]$ and $NoP = [10, 100, 500, 1000]$ and $R = 10$ runs using total degree and RWR statistic for GNT testing, and RWR and shortest path for GNA testing. Experimental results show that 500 permutations are sufficient to obtain a stable null distribution, regardless of the geneset size (see Fig. 2, 3, 4, 5).

1.3 Geneset network association bootstrap procedures

We hereby explain how null distributions are generated for the geneset network association (GNA) tests, which give an estimate of the strength of interaction between two genesets, S_1, S_2. When we generate a null distribution by sampling two random genesets of size S_1 and S_2, we are performing a test under the null hypothesis of no difference between the strength of association observed for S_1 and S_2 and any two random genesets of equal size. Conversely, when one of the geneset is a Gene Ontology (GO) or pathway term T, it is advisable to be more conservative; in this case, we resample just the input geneset and keep the term T fixed, such that we perform a test under the null hypothesis that there is no difference in strength of interaction between the input geneset and any other random geneset of the same size as term T.

1.4 TCGA data retrieval and preprocessing

We downloaded six dataset from The Cancer Genome Atlas (TCGA) or the Genotype-Tissue EXpression (GTEX) repository using the TCGAbiolinks package [2]. Indeed, sometimes, TCGA data lack of control samples, in those cases we resorted to the data of the Recount2 project [3], that has reprocessed all TCGA and GTEX tissues. For each RNA-seq experiment we download the HTSeq- Counts and proceed to normalise them.

Details on the used datasets are reported in Table 1.

Then a differential expression analysis (DEA) is performed using the edgeR negative binomial generalized log-linear model and FDR correction is applied [4].

We then mapped EntrezID of significant genes to HUGO symbol using the R ORG.HS.EG.DB package, in order to have consistent nomenclatures with network data.

References

1. Menche, J., Sharma, A., Kitsak, M., Ghiassian, S.D., Vidal, M., Loscalzo, J., Barabási, A.L.: Uncovering disease-disease relationships through the incomplete interactome. Science **347**(6224), 841 (2015)
2. Colaprico, A., Silva, T.C., Olsen, C., Garofano, L., Cava, C., Garolini, D., Sabedot, T.S., Malta, T.M., Pagnotta, S.M., Castiglioni, I., *et al.*: Tcgabiolinks: an r/bioconductor package for integrative analysis of tcga data. Nucleic acids research **44**(8), 71–71 (2015)
3. Collado-Torres, L., Nellore, A., Jaffe, A.E.: recount workflow: Accessing over 70,000 human rna-seq samples with bioconductor. F1000Research **6** (2017)
4. Robinson, M.D., McCarthy, D.J., Smyth, G.K.: edger: a bioconductor package for differential expression analysis of digital gene expression data. Bioinformatics **26**(1), 139–140 (2010)

TCGA Study	Cases	Tumor Tissue	Control Study	Controls	Control Tissue	Processing pipeline
BLCA	414	Bladder	TCGA	19	Bladder	GDC
BRCA	1102	Breast	TCGA	113	Breast	GDC
DLBC	48	Lymph nodes	GTEX	595	Blood	Recount
LAML	113	Bone marrow	GTEX	595	Blood	Recount
LUSC	502	Lung	TCGA	49	Lung	GDC
PRAD	498	Prostate	TCGA	52	Prostate	GDC

Table 1 RNA sequencing datasets used for the GNA analysis. For each dataset, we report the TCGA code, the number of cases, the tumor tissue, the study and the number of control samples, control tissue and the RNAseq processing pipeline used for quantification for both cases and controls.

2 Supplementary Figures

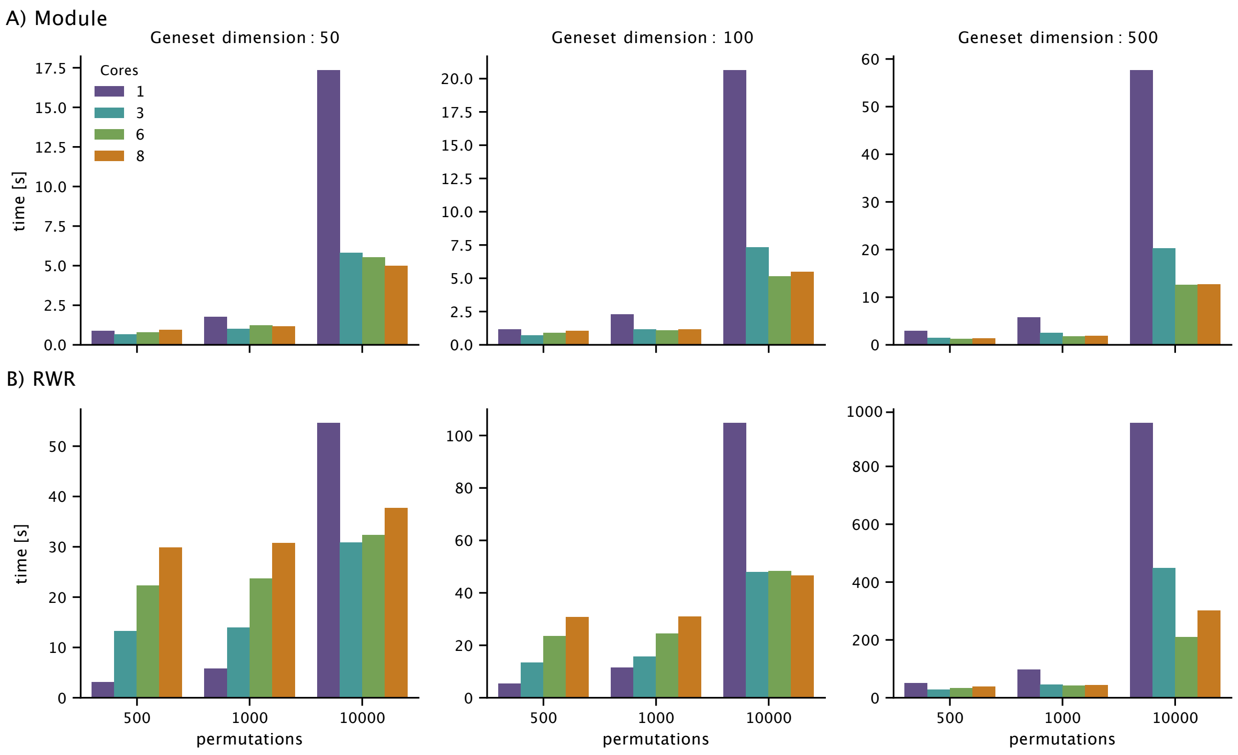

Figure 1 Running time of GNT tests using parallel sampling. We report the number of permutations on the x-axis, the average running time on the y-axis and different hues to denote different number of cores. A) Performing module GNT analysis is considerably faster on all configurations when using at least 2 cores. B) For the RWR GNT, instead, performance improvement is observed only for large genesets or large number of permutations; for few permutations and small genesets, setting up the multi-core architecture introduces a significant overhead not compensated by parallelizing the sampling process.

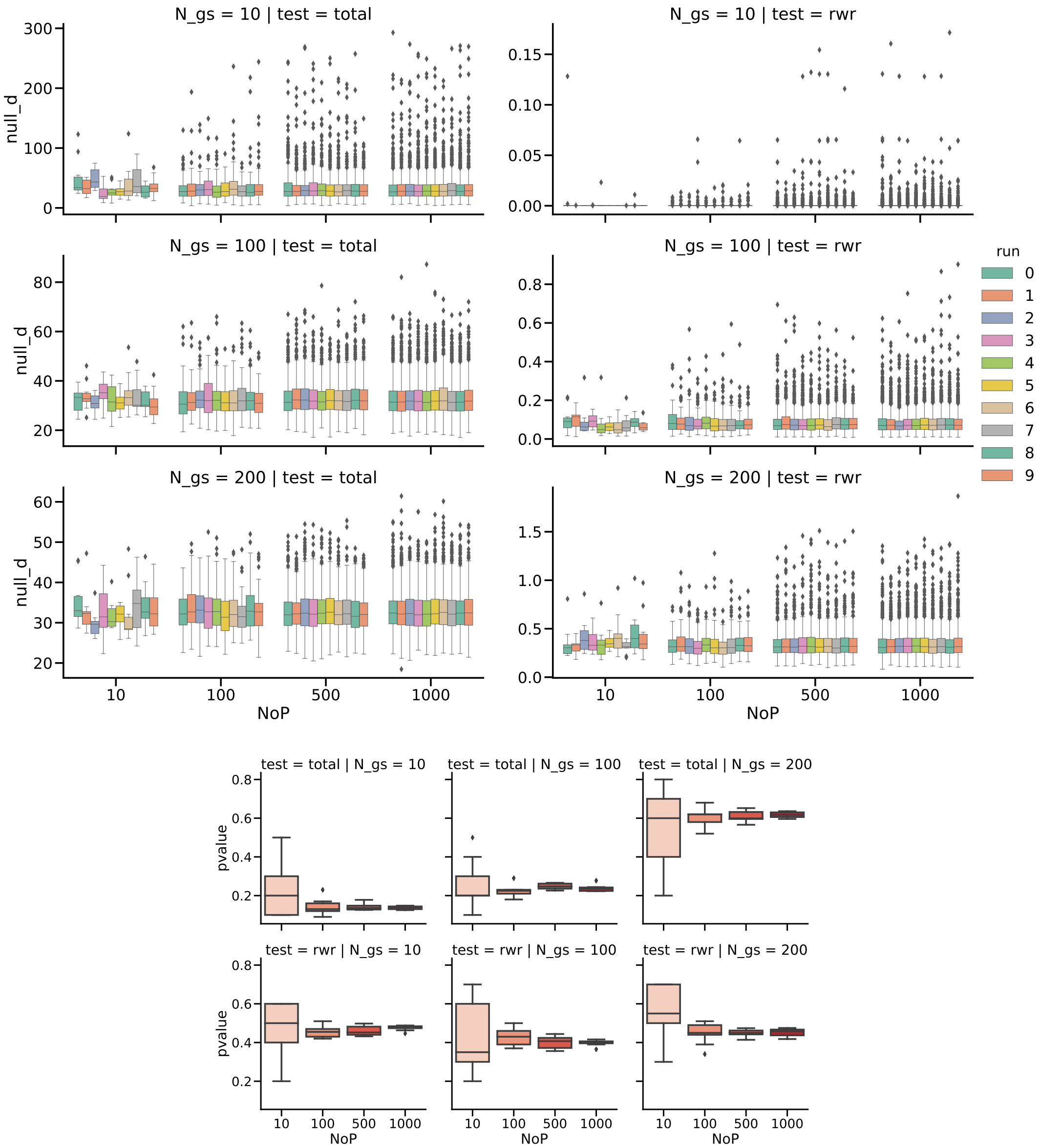

Figure 2 Stability of empirical null distributions for GNT testing on the BioGRID network. A) For each GNT test (columns) and each geneset size (N_gs, rows), we show the box plot of NoP samples of the the null distributions for each run. For a small number of samples, the distribution is relatively unstable, however with more than 100 samples the distributions are stabilized. B) For each GNT test (rows) and each geneset size (columns), we show the box plot of p-values for each run. As the number of permutations increases, the p-value stabilizes as well. Wider box plots reflect the fact that the same observed statistic has different significance levels, since the same geneset is tested for each run. However, for the same geneset we expect all p-values to be the same.

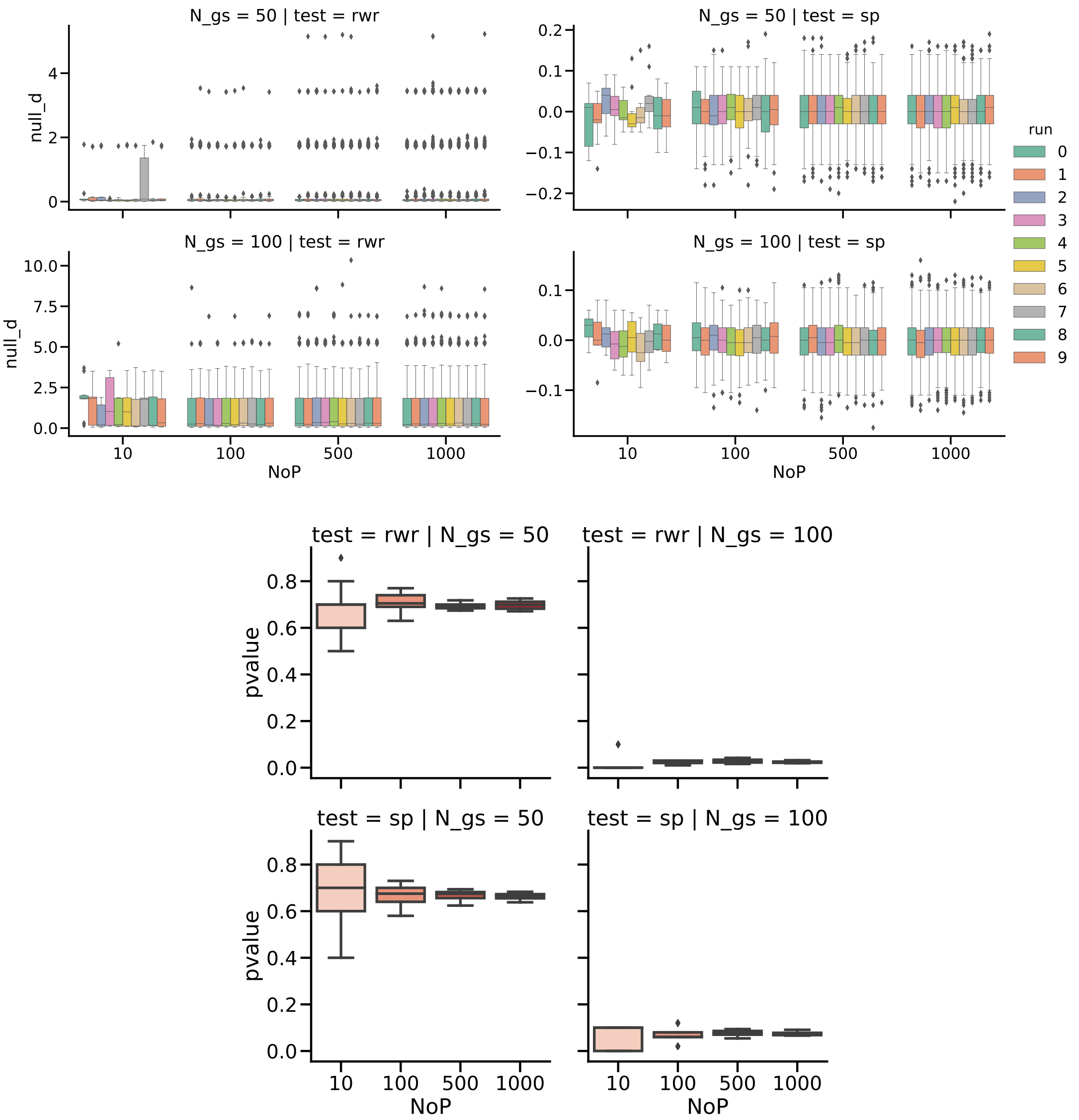

Figure 3 Stability of empirical null distributions for GNA testing on the BioGRID network. A) For each GNA test (columns) and each geneset size (N_gs, rows), we show the box plot of NoP samples of the the null distributions for each run. For a small number of samples, the distribution is relatively unstable, however with more than 100 samples the distributions are stabilized. B) For each GNT test (rows) and each geneset size (N_gs, columns), we show the box plot of p-values for each run. As the number of permutations increases, the p-value stabilizes as well. Wider box plots reflect the fact that the same observed statistic has different significance levels, since the same geneset is tested for each run. However, for the same geneset we expect all p-values to be the same.

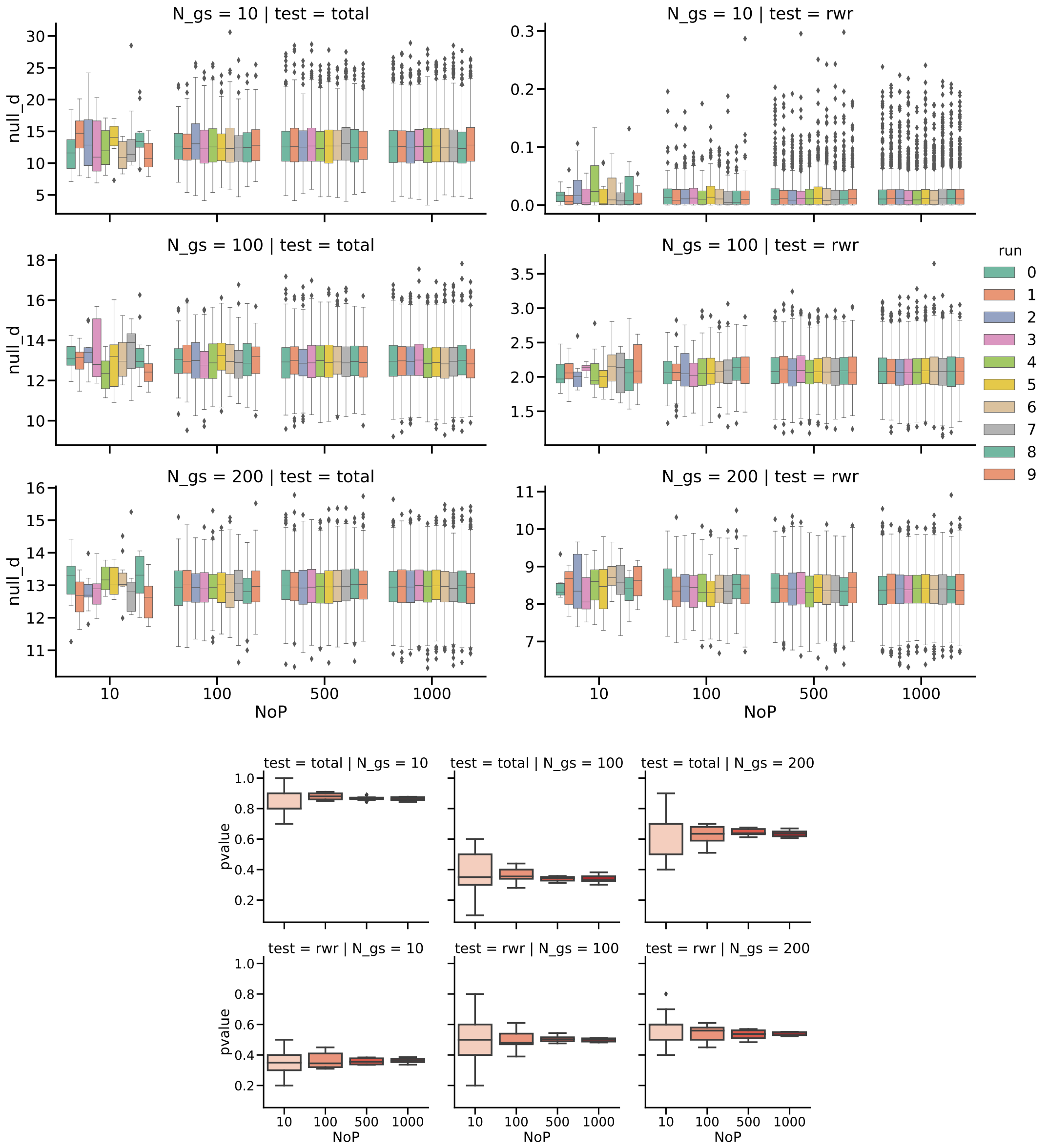

Figure 4 Stability of empirical null distributions for GNT testing on the metabolic network. A) For each GNT test (columns) and each geneset size (N_gs, rows), we show the box plot of NoP samples of the the null distributions for each run. For a small number of samples, the distribution is relatively unstable, however with more than 100 samples the distributions are stabilized. B) For each GNT test (rows) and each geneset size (N_gs, columns), we show the box plot of p-values for each run. As the number of permutations increases, the p-value stabilizes as well. Wider box plots reflect the fact that the same observed statistic has different significance levels, since the same geneset is tested for each run. However, for the same geneset we expect all p-values to be the same.

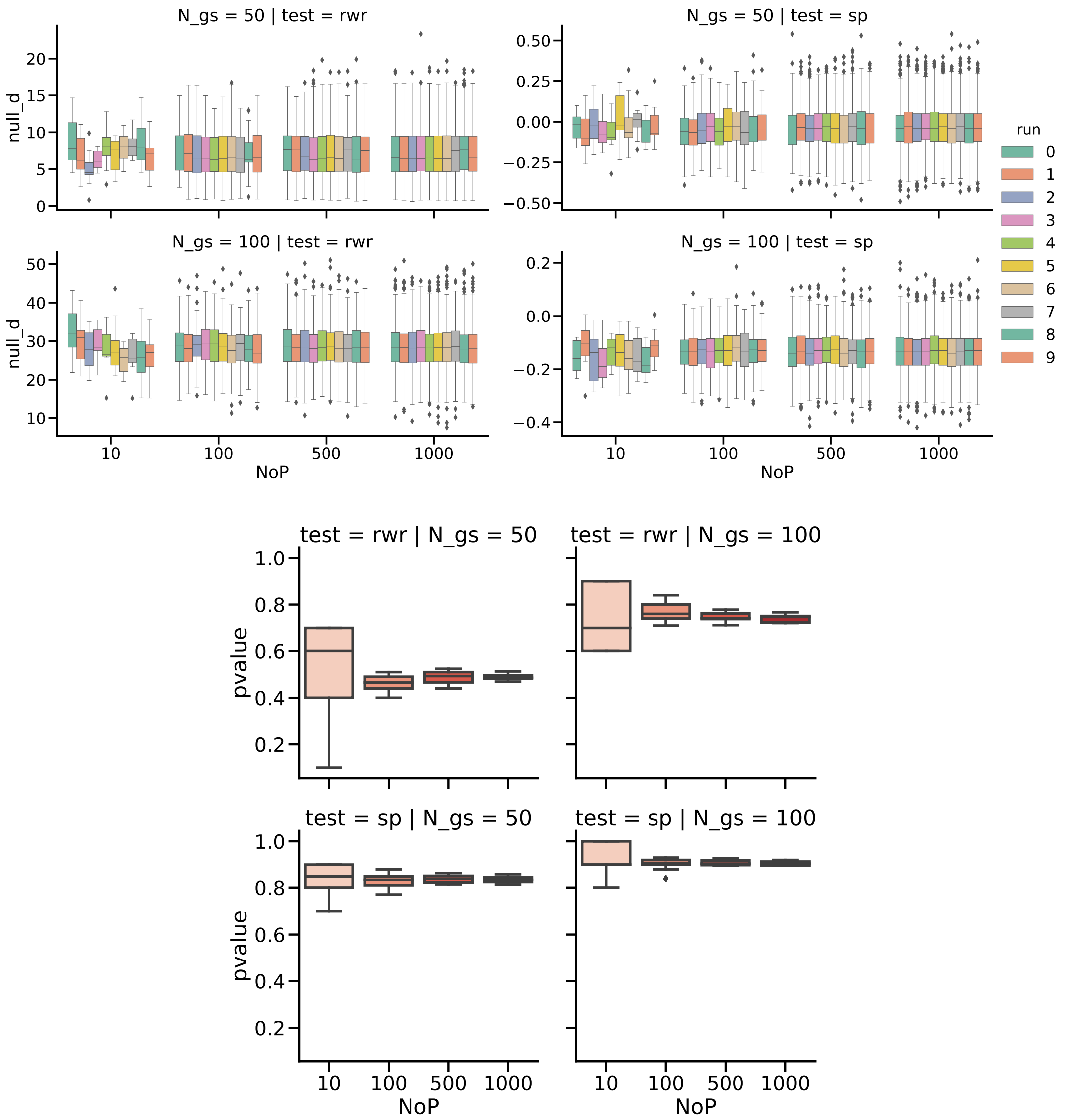

Figure 5 **Stability of empirical null distributions for GNA testing on the metabolic network.** A) For each GNA test (columns) and each geneset size (N_gs, rows), we show the box plot of NoP samples of the the null distributions for each run. For a small number of samples, the distribution is relatively unstable, however with more than 100 samples the distributions are stabilized. B) For each GNT test (rows) and each geneset size (N_gs, columns), we show the box plot of p-values for each run. As the number of permutations increases, the p-value stabilizes as well. Wider box plots reflect the fact that the same observed statistic has different significance levels, since the same geneset is tested for each run. However, for the same geneset we expect all p-values to be the same.

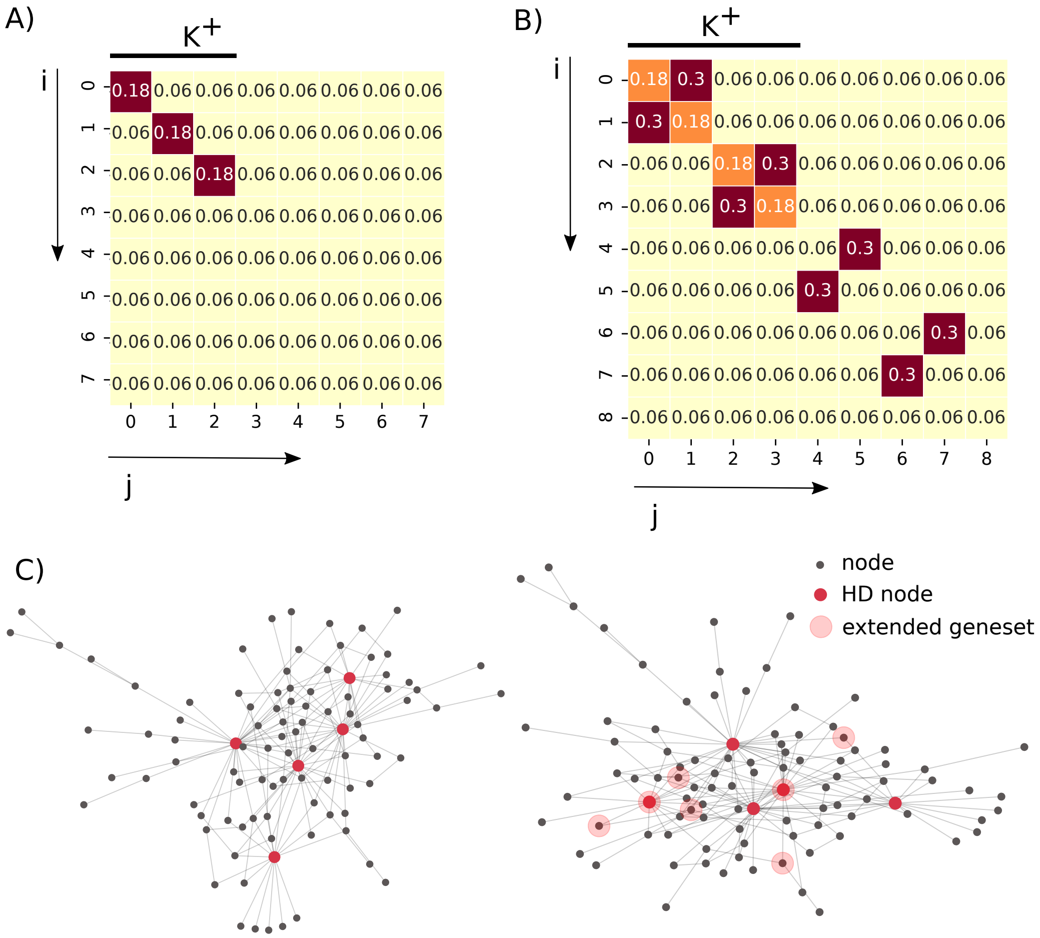

Figure 6 **Generation of synthetic networks** A) Example of a stochastic block model matrix for GNT testing, where each cell reports the value M_{ij}. In this case $p_0 = 0.06$, $\alpha = 3$ and $k^+ = 3$. For benchmarking, $i = 0, 1, 2$ would be considered positive examples, while $i = 4, 5, 6, 7$ would be used as negative ones. B) Example of a stochastic block model matrix for GNA testing, where each cell reports the value M_{ij}. In this case $p_0 = 0.06$, $\alpha = 3$ and $k^+ = 4$ and $\beta = 2$. For benchmarking, $\{0,1\}, \{2,3\}, \{4,5\}, \{6,7\}$ are used to generate mixture genesets. B) Example of the HDN network and geneset generation. First, a network with a number of HDNs (red dots) is created, while all the other nodes have p_0 probability of connection. Then, a geneset is created by taking at random a mixture of HDNs and background nodes (pink nodes).

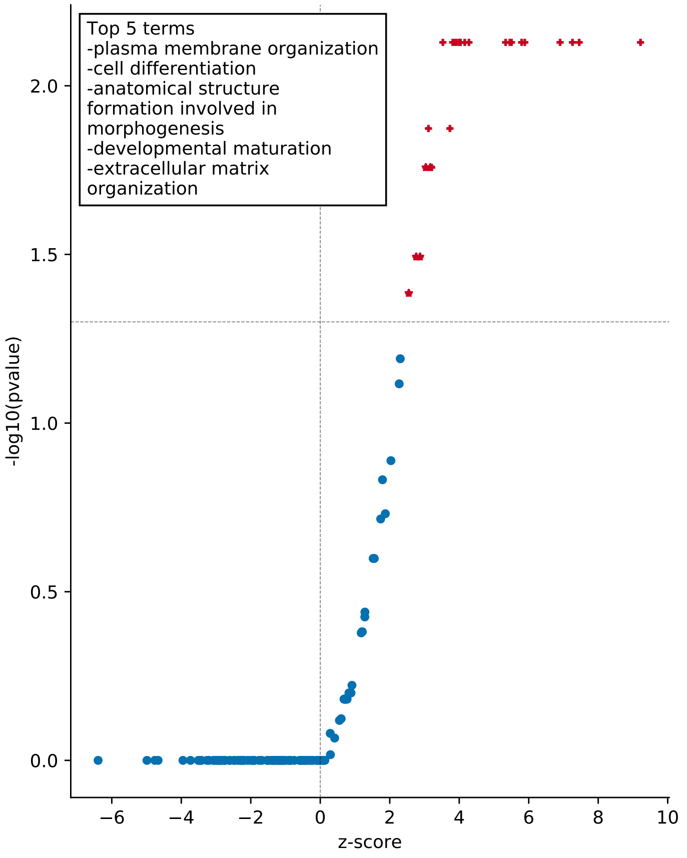

Figure 7 **Results of GNA test for the TCGA-BRCA dataset on the Gene Ontology (GO) slim dataset.** The x-axis reports the absolute value of the z-score of the GNA test statistic under a RWR interaction model, whereas the y-axis reports the $\log_{10}$ adjusted p-values for false discoveries using the Benjamini-Hochberg correction. Terms with adjusted p-value below 0.05 are reported as significant (red dots), with the top 5 marked with a star symbol.
